# Glycocalyx Interactions Modulate the Cellular Uptake of Albumin-Coated Nanoparticles

**DOI:** 10.1021/acsabm.4c01012

**Published:** 2024-10-29

**Authors:** Paulo H. Olivieri Jr, Isabela F. Assis, Andre F. Lima, Sergio A. Hassan, Ricardo J.S. Torquato, Jackelinne Y. Hayashi, Alexandre K. Tashima, Helena B. Nader, Anna Salvati, Giselle Z. Justo, Alioscka A. Sousa

**Affiliations:** † Department of Biochemistry, Federal University of São Paulo, São Paulo, São Paulo 04044-020, Brazil; ‡ Bioinformatics and Computational Biosciences Branch, OCICB, National Institute of Allergy and Infectious Diseases, National Institutes of Health, Bethesda, Maryland 20892, United States; § Department of Nanomedicine & Drug Targeting, Groningen Research Institute of Pharmacy (GRIP), University of Groningen, 9713 AV Groningen, The Netherlands

**Keywords:** nanoparticles, albumin, glycocalyx, heparin, uptake

## Abstract

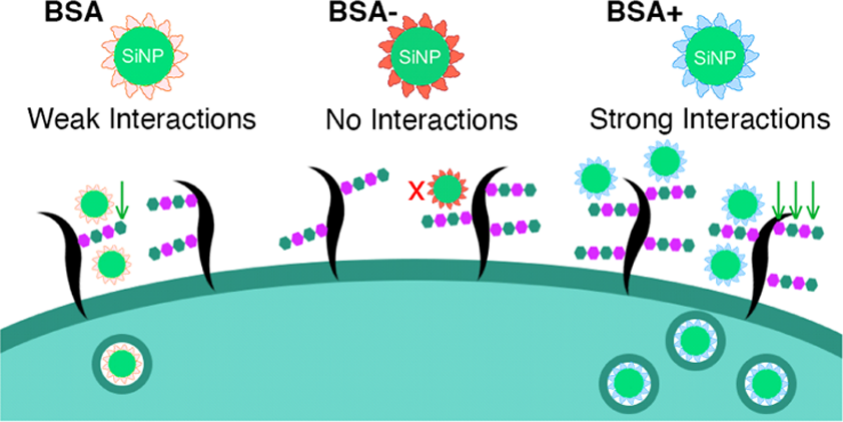

Albumin-based nanoparticles (ABNPs) represent promising drug carriers in nanomedicine due to their versatility and biocompatibility, but optimizing their effectiveness in drug delivery requires understanding their interactions with and uptake by cells. Notably, albumin interacts with the cellular glycocalyx, a phenomenon particularly studied in endothelial cells. This observation suggests that the glycocalyx could modulate ABNP uptake and therapeutic efficacy, although this possibility remains unrecognized. In this study, we elucidate the critical role of the glycocalyx in the cellular uptake of a model ABNP system consisting of silica nanoparticles (NPs) coated with native, cationic, and anionic albumin variants (BSA, BSA+, and BSA−). Using various methodologies–including fluorescence anisotropy, dynamic light scattering, microscale thermophoresis, surface plasmon resonance spectroscopy, and computer simulations—we found that both BSA and BSA+, but not BSA–, interact with heparin, a model glycosaminoglycan (GAG). To explore the influence of albumin-GAG interactions on NP uptake, we performed comparative uptake studies in wild-type and GAG-mutated Chinese hamster ovary cells (CHO), along with complementary approaches such as enzymatic GAG cleavage in wild-type cells, chemical inhibition, and competition assays with exogenous heparin. We found that the glycocalyx enhances the cell uptake of NPs coated with BSA and BSA+, while serving as a barrier to the uptake of NPs coated with BSA–. Furthermore, we showed that harnessing albumin-GAG interactions increases cancer cell death induced by paclitaxel-loaded albumin-coated NPs. These findings underscore the importance of albumin-glycocalyx interactions in the rational design and optimization of albumin-based drug delivery systems.

## Introduction

Engineered nanoparticles (NPs) show great promise in delivering potent therapeutic drugs to target cells within tumors. Among these, albumin-based NPs (ABNPs) stand out for their versatility and biocompatibility.^1−6^ They offer nonimmunogenic and nontoxic characteristics, easy surface conjugation, versatile drug carrier capabilities, and enhanced tumor accumulation through binding to endothelial (gp60) and tumor-associated receptors (e.g., SPARC).^1−3,5,7−10^ Both native and cationized albumin variants have been extensively utilized in various drug delivery and gene therapy applications.^1,4,11−18^

Understanding how ABNPs are taken up by cells is crucial for improving their effectiveness as drug delivery vehicles. While mechanistic studies of ABNP uptake generally focus on receptor binding and entry pathways,^19−23^ this perspective remains incomplete, as it overlooks the potential involvement of the cellular glycocalyx in mediating NP-cell interactions.^24,25^

The glycocalyx is a dense layer of glycoproteins and proteoglycans that coats the outer surface of cells, serving as a dynamic interface between the cell and its environment.^26−28^ This structure is highly negatively charged due to the presence of proteoglycans, which consist of proteins covalently bound to glycosaminoglycans (GAGs) such as heparan sulfate (HS) and chondroitin sulfate (CS), as well as hyaluronic acid chains.^29,30^ The glycocalyx can function as a molecular sieve, preventing large molecules and NPs, particularly those with anionic properties, from reaching the plasma membrane and becoming internalized.^25,31−37^ For example, we recently showed that the glycocalyx of Chinese hamster ovary (CHO) cells can function as a charge-based barrier against the internalization of anionic polystyrene NPs.^35^ However, regarding the glycocalyx as a mere passive physical barrier against the cell entry of macromolecules, pathogens, or NPs would be inaccurate.^38−44^ Thanks to its complex chemistry, flexible architecture, and interaction networks, the glycocalyx is now recognized as an organelle actively engaged in a myriad of cellular processes.^26,27,45,46^ In the context of engineered NPs utilized for drug delivery, recent studies have revealed that distinct components of the glycocalyx can serve as primary receptors, binding to specific proteins within the NP corona, and facilitating NP internalization into the cell.^47,48^

Notably, albumin interacts with the cellular glycocalyx, a phenomenon particularly studied in endothelial cells.^49−51^ In vivo, albumin protects the endothelial glycocalyx against shedding, thereby playing a pivotal role in maintaining vascular integrity and permeability.^49,52−55^ Albumin-glycocalyx interactions have been speculated to arise from electrostatic interactions between the negative charges on GAGs and positively charged patches on albumin.^49^ Overall, these observations suggest that the cell glycocalyx may play a significant role in modulating the endocytosis of ABNPs, although this possibility remains unrecognized.

In this study, we investigated how the glycocalyx controls the cellular uptake of ABNPs. Our primary system consisted of 50 nm silica NPs coated with native, cationic, and anionic albumin variants. This model system provided a well-controlled setup with defined size, uniformity, and surface properties. Additionally, albumin-coated NPs hold significant interest as drug delivery vehicles for cancer therapy.^6,56−59^ Through experimental and computational approaches, we found that both native and cationic albumin interact with heparin, a model GAG, while such interactions are absent in the case of anionic albumin. Moreover, cell uptake experiments revealed that the glycocalyx can either facilitate or hinder NP internalization depending on the type of albumin coating.

## Results and Discussion

### Native, Anionic, and Cationic Albumin

**Figure 1:**
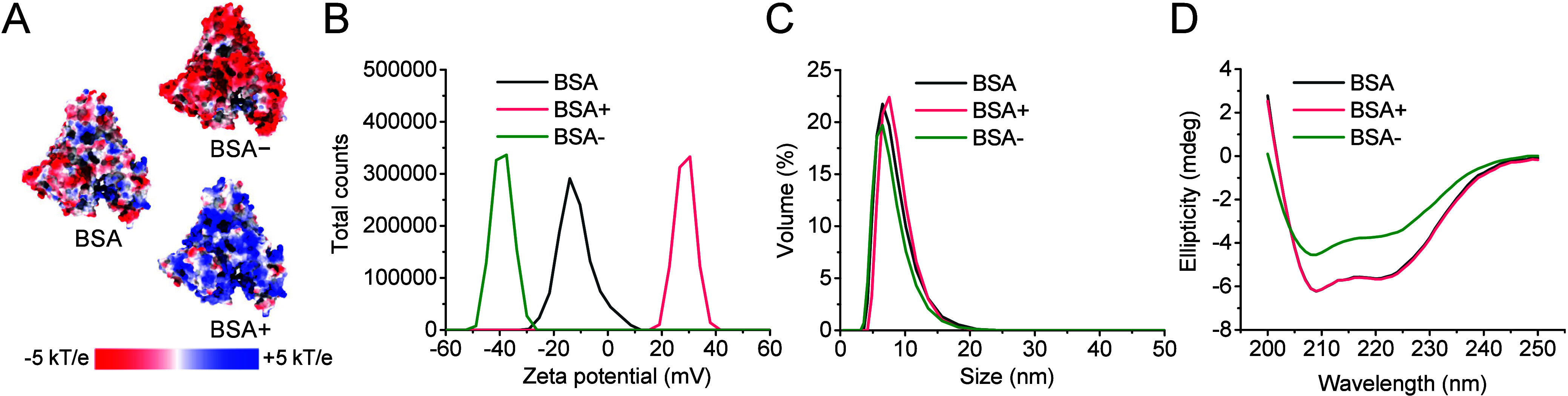
Characterization of BSA, BSA+, and BSA–. (A) Electrostatic surface potential of native BSA (total charge −15 e), BSA+ (total charge +20 e) and BSA– (total charge −40 e). (B) Zeta potential, (C) dynamic light scattering, and (D) CD spectroscopy characterization of BSA, BSA+, and BSA–.

We employed native bovine serum albumin (BSA), cationic albumin (BSA+), and anionic albumin (BSA−) for coating fluorescent silica NPs of 50 nm in size. Cationization of BSA was achieved by converting surface carboxyl groups into positively charged amino groups, whereas anionization (succinylation) involved converting surface amino groups into carboxyl groups. Figure 1A presents the electrostatic surface potential of native BSA and the modified potentials for BSA+ and BSA–; the latter were calculated by altering the most surface-exposed carboxyl and amino groups, and are intended for illustrative purposes only. Zeta potential (ZP) measurements confirmed these charge modifications, yielding approximately −13 mV for BSA, + 30 mV for BSA+, and −40 mV for BSA– (Figure 1B). Hydrodynamic diameter (HD) was determined by dynamic light scattering (DLS), showing no significant differences between the variants (HD ∼ 7 nm) (Figure 1C). Circular dichroism spectroscopy (CD) analysis revealed no apparent change in protein secondary structure between BSA and BSA+ (Figure 1D). However, the CD spectrum of BSA– displayed visible structural alterations, consistent with previous reports indicating partial denaturation of BSA upon succinylation.

### Experimental Analysis of Albumin-Heparin Interactions

**Figure 2:**
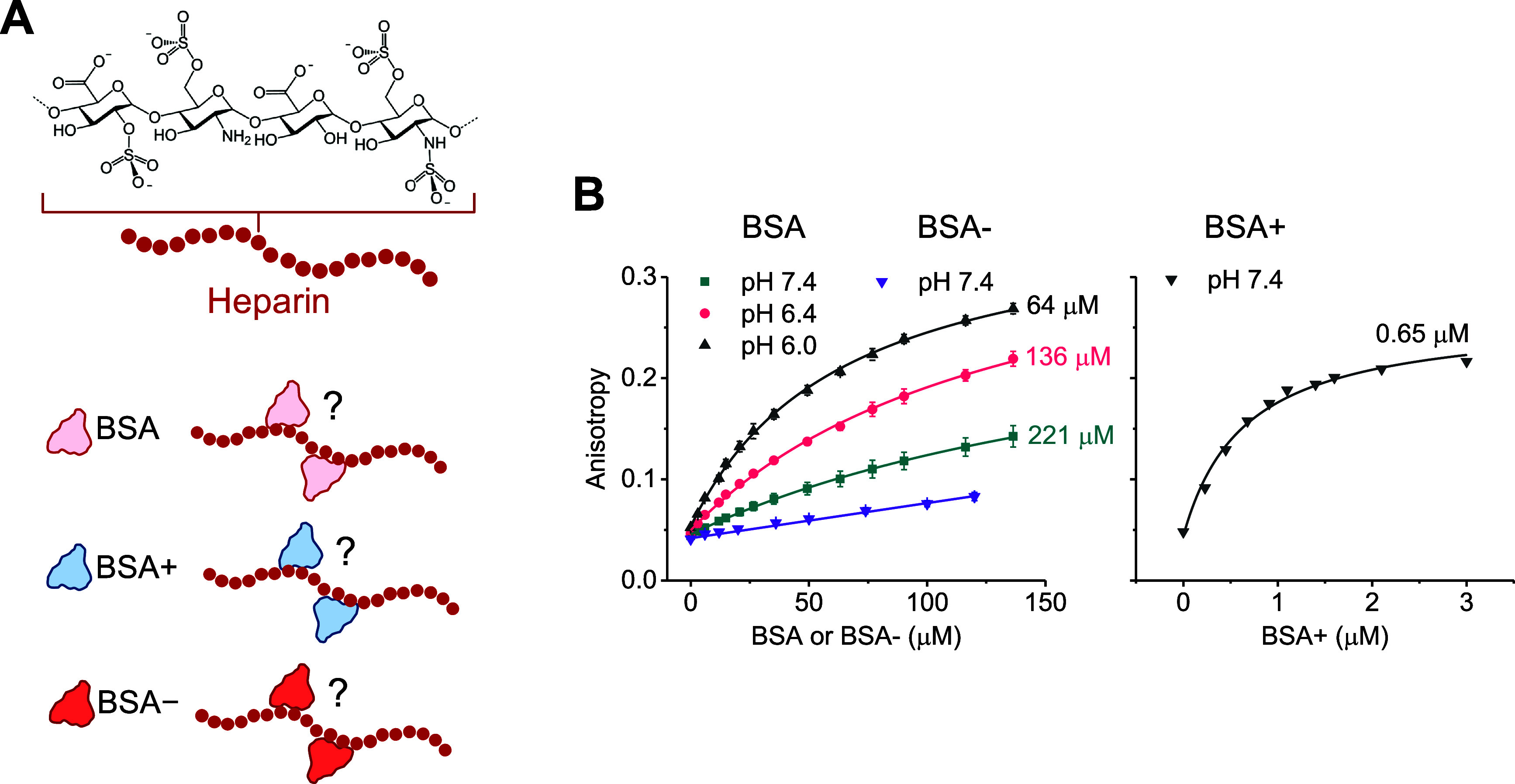
Characterization of albumin-heparin interactions. (A) Schematic illustration of heparin molecule with interacting BSA, BSA+, and BSA– proteins. (B) Fluorescence anisotropy measurements of fluorescein-labeled heparin upon titration with BSA, BSA+ and BSA– at the indicated pH values. Solid lines represent fits to the data using the equation described in the Materials and Methods Section. Apparent values of binding affinity (*K*_D_) are annotated near each fit. Curves represent the average of three independent measurements.

Native albumin has been reported to interact with the endothelial glycocalyx.^49−51,54^ It has been theorized that this could be mediated by electrostatic forces between the anionic GAGs and positively charged patches on albumin.^49^ To our knowledge, however, a direct assessment of albumin-GAG interactions has not been conducted to date (Figure 2A). Therefore, we sought to address this gap before evaluating NP-GAG interactions and NP uptake. For this purpose, we used a combination of experimental and computational approaches to investigate the binding of native BSA to heparin, a model GAG. Binding experiments were also conducted for BSA+ and BSA–, which were anticipated to exhibit stronger and weaker interactions with heparin, respectively.

We employed fluorescence anisotropy to measure the interactions. Anisotropy measurements are advantageous for being unaffected by sample dilution and first-order inner-filter effects. We first titrated BSA into a solution of fluorescein-labeled heparin and monitored the corresponding changes in anisotropy. These titrations were conducted at varying pH levels, including pH 6.0, 6.4, and 7.4. In the absence of BSA, the anisotropy was ∼0.05, consistent with previous findings.^60^ The addition of BSA led to a dose-dependent increase in anisotropy, implying complexation (Figure 2B). The results were used to derive an apparent equilibrium dissociation constant (*K*_D_), yielding 221 μM at pH 7.4. Although indicative of very weak interactions, this may hold physiological significance given the large plasmatic concentration of albumin, which ranges from 500 to 750 μM. This weak binding affinity aligns with findings from a prior study employing electron spin resonance to examine the molecular mobility of albumin within the endothelial cell glycocalyx.^50^ It was demonstrated that albumin undergoes transient interactions with the glycocalyx and manifests no persistent binding. Figure 2B further indicated that the binding affinity was enhanced when the pH was below 7.4. Presumably, the reduced negative charge of BSA at lower pH leads to decreased long-range repulsion toward heparin, thus promoting stronger interactions between the two molecules. Additionally, fluorescence anisotropy revealed that the binding affinity between BSA+ and heparin was much stronger as expected, yielding an apparent *K*_D_ of 0.65 μM (Figure 2B). In contrast, anisotropy measurements indicated lack of significant interactions between heparin and BSA– at pH 7.4 (Figure 2B).

### Computational Analysis of Albumin-Heparin and Albumin-Heparan Sulfate Interactions

**Figure 3:**
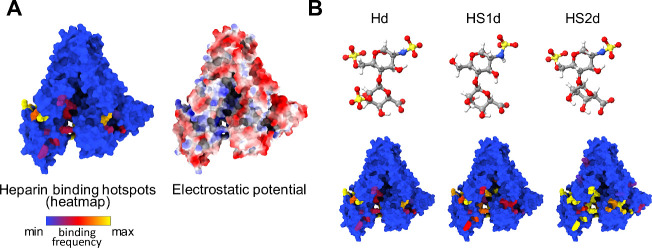
Binding regions of heparin and heparan sulfate disaccharides to native albumin. (A) Heatmap of heparin binding frequencies obtained from multiple MD simulations and corresponding surface electrostatic potential. (B) Ball-and-stick representation of the structure of heparin (Hd), heparan monosulfated (HS 1d), and heparan disulfated (HS 2d) disaccharides and the corresponding binding spots on albumin.

To gain molecular-level insights into native BSA-GAG interactions, we conducted computer simulations with model systems. Molecular dynamics (MD) simulations show that heparin disaccharides (Hd) are attracted to local regions of positive electrostatic potential on the albumin surface. Once Hd and albumin are close enough, binding is fine-tuned through H-bond interactions between lysine (K) or arginine (R) residues and the *SO*_3_^-^ or *CO*_2_^-^ groups in Hd; the sugar rings help to further stabilize binding through hydrophobic interactions with nonpolar moieties, including the methylene groups of K and R side chains. Therefore, binding of Hd to albumin is restricted to a limited number of residues at specific locations on the surface. Hd is most attracted to R434 (numbering from UniProt AC P02768), while all other associations seem to be transient, judging by the frequency of the interactions (Figure 3A). In the heatmaps of Figure 3, binding frequencies below 10% are marked in blue, while those above 25% are highlighted in yellow. For reference, R434 has a binding frequency greater than 80%. Overall, these findings underscore the significance of the orientation and conformation of adsorbed albumin, where misoriented or denatured albumin may compromise the optimal interaction with surface GAGs.

In the heparin polymer, each Hd unit would be attracted to the same hotspots identified above. As shown in Figure 3A, the number of hotspots is relatively small, so a longer polymer would not necessarily have more interactions with albumin, since available sites will get saturated. Nonetheless, several albumins can bind to the same polymer, since NMR and other structural experiments have shown that heparin adopts extended conformations in solution.

Comparative analysis shows that albumin attracts Hd and heparan sulfate disaccharides (monosulfated, HS 1d, and disulfated, HS 2d) largely to the same spots on its surface but with different strengths (Figure 3A, B). Hd and HS 1d appear to bind similarly despite HS 1d having a lower negative charge (−2 e) than Hd (−4). Although Hd has more H-bond-forming groups (*SO*_3_^-^ and *CO*_2_^-^ in inset Figure 3) to bind to K and R, it is also less attracted to albumin due to its larger overall negative charge; by contrast, HS 1d has fewer H-bonding groups, but its charge is also less negative. The similarity in binding thus stems from a compensation of attractive/repulsive forces. This balance appears to break down for HS 2d. Compared to Hd and HS 1d, HS 2d has an intermediate overall charge (−3) and number of H-bonding groups but binds albumin with higher strength than either ligand (Figure 3B). Because trisulfated (HS 3d) has an overall charge equal to that of Hd (−4) and tetrasulfated (HS 4d) is even more negative (−5), as a function of sulfation level, HS 2d might optimize binding efficiency. Therefore, given the variety of HS species in the cell, measured properties mediated by HS polymers (here, albumin binding to the glycocalyx) would be an average weighted by the prevalence of each type.

### Preparation of Albumin-Coated NPs

**Figure 4:**
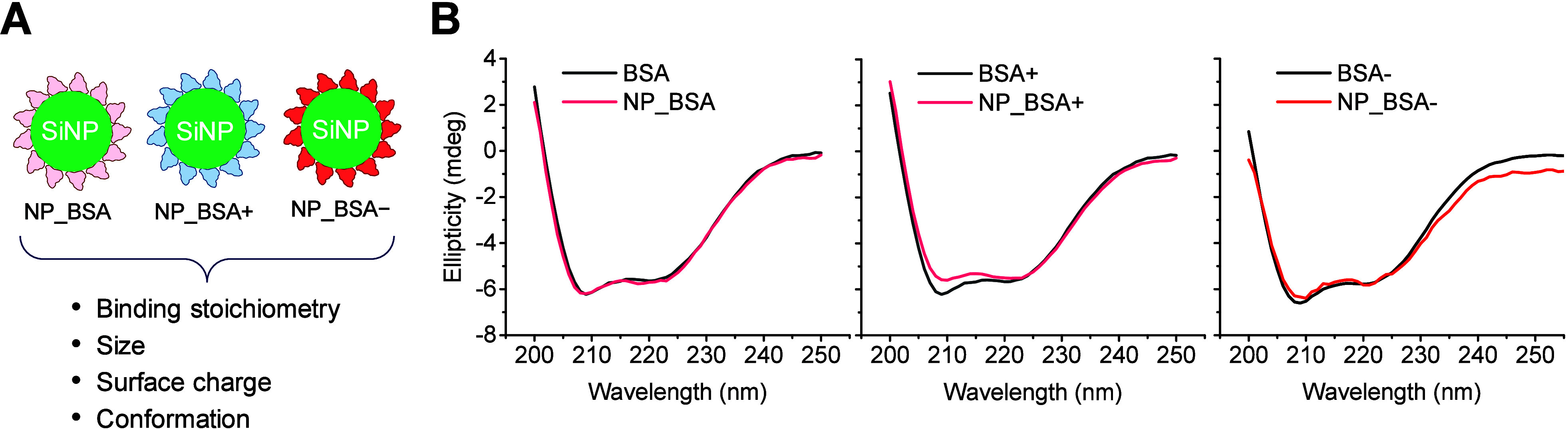
Characterization of albumin-coated NPs. (A) Schematic illustration of NP_BSA, NP_BSA+, and NP_BSA–, and relevant parameters for their characterization. (B) CD spectroscopy characterization of adsorbed BSA, BSA+, and BSA– compared to the free proteins.

Having characterized the interactions between heparin and albumin, we deposited the various albumin variants onto a silica core to create a model of ABNPs with controlled size and uniformity. We characterized the coated NPs (NP_BSA, NP_BSA+ and NP_BSA−) in terms of their binding stoichiometry, overall size, surface charge, and protein conformation (Figure 4A). DLS and ZP measurements confirmed successful NP coating (Table 1). Determination of the binding stoichiometry yielded between 230 and 350 BSA molecules per NP (Table 1), which is in excellent agreement with the theoretical prediction of 200 molecules per NP based on geometric considerations (refer to the Materials and Methods section). These findings suggest that the observed larger HD for NP_BSA+ (around 102 nm) is unlikely to be caused by multilayer formation of BSA+; instead, it is more likely attributed to inherent limitations of the DLS technique,.e.g., in handling more heterogeneous size distributions.^61,62^ Results from CD spectroscopy showed virtually no difference in the secondary structure between adsorbed and free BSA, while BSA+ and BSA- exhibited some structural change after adsorption (Figure 4B). The preservation of the overall BSA structure following adsorption onto silica NPs is consistent with the outcomes of computer simulations,^63^ suggesting that the native functionality of BSA (e.g., receptor binding) is retained.

**Table 1:** Binding Stoichiometry (*n*, #BSA molecules per NP), Hydrodynamic Diameter (HD), Polydispersity Index (PDI), and ZP Measurements of Bare and Various Coated NPs (*n* = 3)

NPs	*n*	HD (nm)	PDI	ZP (mV)
Bare	-	46 ± 1	0.08	–32 ± 9
BSA	243	61 ± 1	0.15	–19 ± 3
BSA+	230	102 ± 14	0.28	+4 ± 1
BSA–	350	59 ± 15	0.27	–45 ± 9
FBS	-	84 ± 10	0.16	–30 ± 4

### Heparin Binds to Albumin-Coated NPs

**Figure 5:**
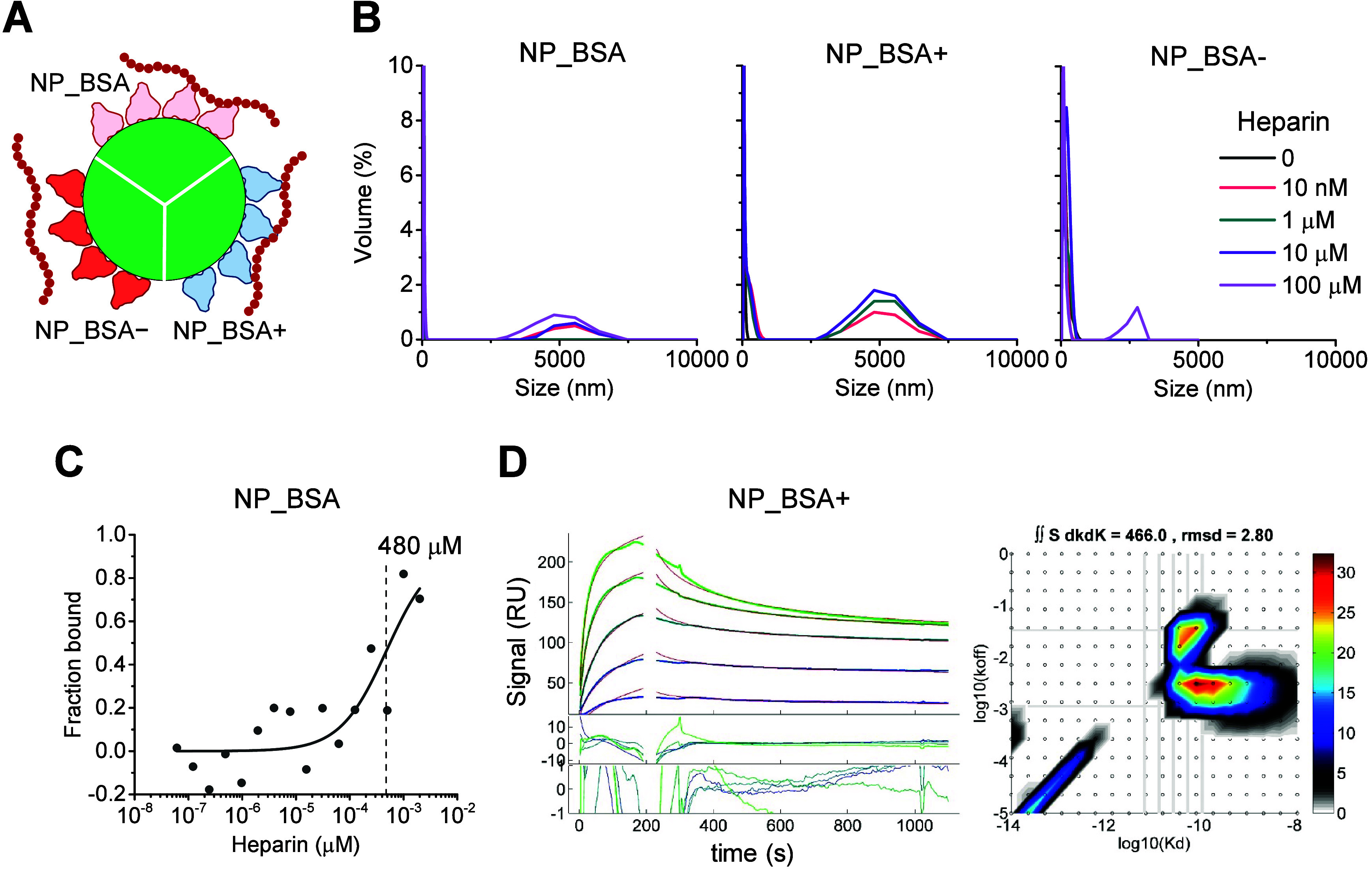
Characterization of heparin-NP interactions. (A) Schematic illustration of albumin-coated NP with interacting heparin molecules. (B) Dynamic light scattering measurements of NP_BSA, NP_BSA+, and NP_BSA– titrated with heparin. (C) Microscale thermophoresis measurements of NP_BSA titrated with heparin. Points represent the average of three measurements. The apparent binding affinity (*K*_D_) is indicated in the plot. (D) Surface plasmon resonance characterization of NP_BSA+ interactions with heparin. Left panel: experimental traces (green and blue lines), best-fit curves (red lines), and fitting residuals. Right panel: calculated affinity and rate constant distributions. Two primary binding modes are observed with characteristic *K*_D_ values in the sub-nM range and *k*_off_ values around 10^–2^ and 10^–3^ s^–1^.

Having established that heparin binds to both BSA and BSA+, but not BSA–, we next investigated its binding to the respective coated NPs (Figure 5A). Initially, we employed DLS measurements to evaluate the occurrence of heparin-NP interactions. The experiments consisted in recording DLS data for the NPs in the presence of increasing concentrations of heparin. The results indicated that heparin induced the formation of large aggregates for both NP_BSA and NP_BSA+, with sizes ranging from 3 to 7 μm (Figure 5B). We conclude that heparin interacted favorably with the BSA and BSA+ coatings. These interactions bridged NPs, leading to their aggregation. In contrast, NP_BSA– showed no signs of aggregation, except at the highest heparin concentration, consistent with the absence of detectable interactions between BSA– and heparin as demonstrated by fluorescence anisotropy (Figure 2B). Next, we employed microscale thermophoresis (MST) as a complementary technique to evaluate the binding between NP_BSA and heparin. For this purpose, we titrated NP_BSA with heparin and recorded the corresponding MST signal following each titration (Figure 5C). Although the obtained data were noisy, they suggested weak interactions. The apparent binding affinity was estimated as *K*_D_ ∼ 480 μM; however, this value must be interpreted with caution due to NP aggregation. A similar analysis could not be performed for NP_BSA+ due to poor data quality, likely caused by pronounced NP aggregation in the presence of heparin. To further investigate the interactions between heparin and NP_BSA+, we employed surface plasmon resonance (SPR) spectroscopy, a surface-based technique. The experiment involved immobilizing heparin onto a sensor surface and flowing NP_BSA+ over it, thereby approximating the configuration encountered during NP interactions with cell-surface GAGs. However, due to technical limitations, we were unable to titrate a sufficiently large concentration of NP_BSA+ to saturate the immobilized heparin. Nonetheless, interactions between NP_BSA+ and heparin were observed (Figure 5D). Two major binding modes were detected with an estimated *K*_D_ in the sub-nM range and *k*_off_ rate constants in the range from 3 × 10^–3^ to 2 × 10^–2^ s^–1^. These low *k*_off_ values imply that NP_BSA+ remained strongly bound to the surface during the dissociation phase, possibly due to avidity effects (a single coated NP binding to multiple heparin molecules). This observation raises the possibility that avidity effects may be operational at the cell surface. A similar analysis for NP_BSA was not possible due to its weak binding affinity toward heparin.

Collectively, the above biophysical characterization revealed weak binding between heparin and native BSA. Heparin also interacted favorably with the BSA coating on silica NPs. Moreover, and as anticipated, heparin exhibited stronger binding to the BSA+ coating. In contrast, heparin showed no significant binding to either free BSA– or the BSA– coating.

### Glycocalyx Interactions Enhance the Cellular Uptake of NP_BSA and NP_BSA+

**Figure 6:**
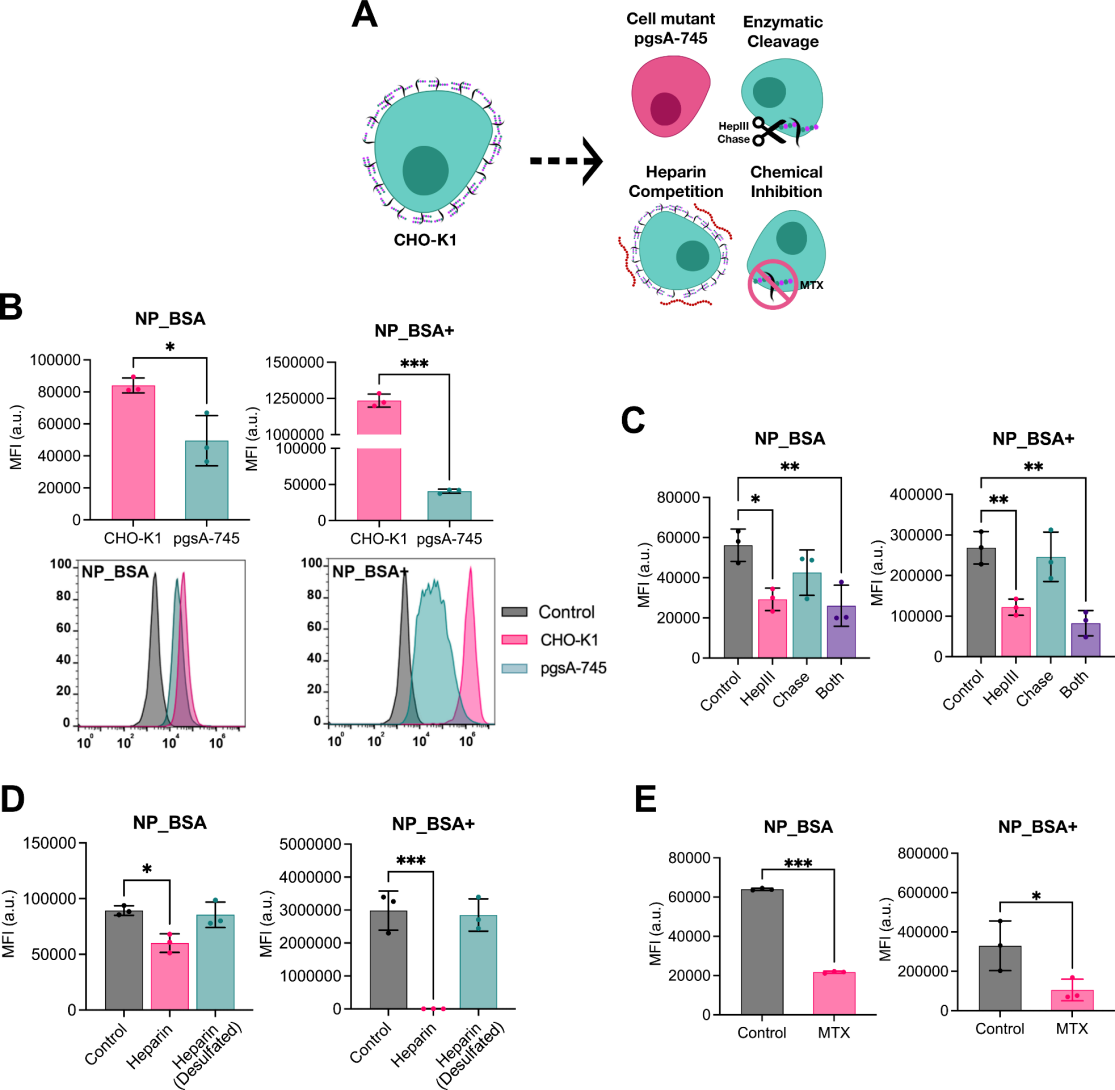
Uptake of NP_BSA and NP_BSA+ by CHO cells. (A) Schematic representation of methods used to evaluate the impact of the cell glycocalyx on NP uptake. CHO-K1 and pgsA-745 cells were incubated with NPs (3 nM) for 4 h in culture medium, washed to remove excess particles, then analyzed by flow cytometry. In another set of experiments, CHO-K1 cells were pretreated with glycosidic enzymes, excess heparin, or chemical inhibitors before NP administration for 2 h in culture medium. (B) NP uptake by CHO-K1 and pgsA-745 cells. (C) NP uptake by CHO-K1 cells treated with HepIII, Chase, or both. (D) NP uptake by CHO-K1 cells in the presence of excess heparin and desulfated heparin. (E) NP uptake by CHO-K1 cells treated with MTX.

As a next step, we employed a variety of complementary methods to explore how interactions between NP_BSA/NP_BSA+ and the glycocalyx regulate NP cell uptake. The various methods, detailed below, entailed the use of GAG-mutated cells, enzymatic cleavage of GAGs, chemical inhibitors, and competition assays (Figure 6A). Individually, each analysis method may not conclusively determine how the glycocalyx affects NP uptake, as potential interference with other cellular processes could confound data interpretation. However, when combined, these methods can provide a robust and reliable picture concerning the glycocalyx’s role in NP uptake.

Our primary model system consisted of wild-type CHO-K1 cells and the xylosyltransferase-deficient mutant pgsA-745 cell line.^64^ CHO-K1 cells possess approximately equal levels of HS and CS, whereas pgsA-745 cells lack both HS and CS (with <5% GAG expression compared to CHO-K1). The basic experimental setup involved exposing cells to NP_BSA and NP_BSA+ for either 2 or 4 h at 37 °C in serum-free culture medium, followed by flow cytometry analysis. Employing serum-free conditions enabled a more focused examination of the effects of albumin-GAG interactions on uptake without interference from excess serum proteins. We also ensured that the concentrations of NPs and other compounds used were nontoxic to cells (Suppl. Table S1 and Suppl. Figure S1). As a control, we conducted additional uptake experiments at 4 °C to confirm the occurrence of actual NP internalization at 37 °C via energy-dependent endocytosis (Suppl. Figure S2).

First, we notice that the cell uptake of NP_BSA+ by CHO-K1 cells was approximately 15-fold greater than that of NP_BSA, supporting the notion that cationic NPs are endocytosed more efficiently than anionic ones (Figure 6B, compare pink bars). Conversely, in pgsA-745 cells, the difference in uptake levels between NP_BSA+ and NP_BSA was much less pronounced. Most importantly for our discussions, both NP_BSA and NP_BSA+ exhibited reduced endocytosis in the GAG-deficient cell line, with NP_BSA experiencing a 1.7-fold decrease in uptake and NP_BSA+ a significant 30-fold reduction in uptake (Figure 6B).

It is conceivable that the wild-type and mutant CHO cells could exhibit distinct endocytic phenotypes unrelated to surface GAGs, potentially influencing the results outlined in Figure 6B and complicating interpretation. Therefore, we performed additional experiments where we directly compared NP internalization between pristine and enzyme-treated CHO-K1 cells. The enzymes included heparinase III (HepIII) and chondroitinase AC (Chase), which cleave HS and CS, respectively. Enzyme activity was confirmed through immunofluorescence in separate control experiments using anti-HS and anti-CS antibodies (Suppl. Figure S3). We found that CS shedding did not affect NP uptake, whereas shedding of HS reduced uptake for both NP types (Figure 6C). This suggests a particularly important role for HS in mediating uptake, consistent with previous findings for uncoated cationic polystyrene NPs.^35^ When combining HepIII and Chase, cell uptake decreased by approximately 2.1-fold for NP_BSA and 3.3-fold for NP_BSA+.

Next, we conducted a competitive cell uptake assay, whereby we exposed CHO-K1 cells to the NPs in the presence of excess heparin. Exposure to heparin and other sulfated compounds like dextran sulfate and heparan sulfate is often used to demonstrate the role of HS in uptake. The results showed a small decrease in the cellular internalization of NP_BSA, while the uptake of NP_BSA+ was entirely abolished (Figure 6D). The significant inhibition of NP_BSA+ uptake in the presence of exogenous heparin likely results from the strong binding affinity between heparin and BSA+. As a control, we repeated the experiments in the presence of desulfated heparin, which did not affect NP uptake.

To expand on these studies, we examined the impact of the anticancer drug mitoxantrone (MTX) on NP uptake. Recently, this molecule has been reported as an inhibitor of HS-dependent endocytosis.^65,66^ MTX was shown to effectively block the cell entry of HS-dependent cargos, such as supercharged GFP (GFP+), polycation-coated DNA, and SARS-CoV-2, while having minimal impact on clathrin-dependent transferrin endocytosis. Although the exact mechanism of action of MTX is not known, it is worth pointing out that it binds to free heparin and HS and appears to directly target cell-surface HS. Here, we independently verified the inhibitory action of MTX on the cell uptake of cationic polystyrene NPs, which were used as a model HS-dependent cargo (Suppl. Figure S4).^35^ Additionally, we confirmed that MTX does not inhibit clathrin-dependent endocytosis, nor does it impair general fluid phase endocytosis or macropinocytosis more specifically (Suppl. Figure S5).^67,68^ In our studies, MTX efficiently inhibited the cell uptake of both NP_BSA and NP_BSA+ (Figure 6E), therefore implying the involvement of HS on NP endocytosis.

To evaluate if tumor cells also reproduce the same events, we conducted additional uptake experiments using HeLa cells as a model system for cancer (Suppl. Figure S6A and S6B). First, we observed that NP uptake in untreated HeLa cells was approximately 10-fold higher for NP_BSA+ than NP_BSA, consistent with findings in CHO-K1. Next, we compared NP uptake between untreated and enzyme-treated cells. The results were similar to those obtained from CHO-K1, that is, removing CS did not impact NP uptake, while shedding of HS reduced the uptake of both particles. Furthermore, a competitive uptake assay in the presence of excess heparin showed reduced uptake for NP_BSA, whereas the uptake of NP_BSA+ was completely abolished, consistent with results observed in CHO-K1 cells.

Taken together, the above findings indicate that the glycocalyx can facilitate, rather than inhibit, the cell uptake of NPs uniformly covered with BSA or BSA+. These results are consistent with recent reports in the literature that showed that rather than being a barrier to uptake, the glycocalyx can mediate interactions as cell-surface receptors and promote NP uptake,^47,48^ similar to observations for several viruses.^65,69,70^

### The Glycocalyx Acts As a Barrier against the Cell Uptake of Bare NPs and NP_BSA–

We also examined the cell uptake of two NP types lacking attractive interactions with GAGs, namely uncoated plain silica NPs and NP_BSA–. Uptake was assessed in both CHO-K1 and pgsA-745 cells similarly as described above. Additionally, control uptake experiments at 4 °C confirmed NP_BSA– internalization at 37 °C via energy-dependent mechanisms (Suppl. Figure S2).

**Figure 7:**
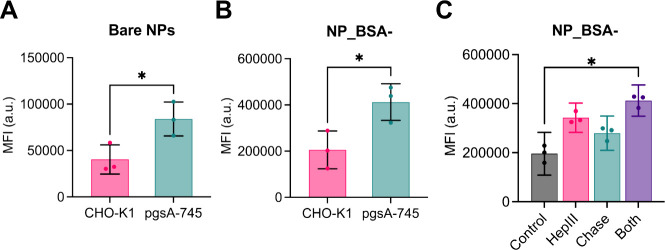
Uptake of (A) bare NPs and (B) NP_BSA– by CHO-K1 and pgsA-745 cells. Cells were incubated with NPs (0.16 nM for bare and 3 nM for NP_BSA−) for 4 h in culture medium, washed to remove excess particles, then analyzed by flow cytometry. (C) Uptake of NP_BSA– by enzyme-treated CHO-K1 cells. The cells were pretreated with glycosidic enzymes before NP administration for 2 h in culture medium.

Interestingly, both bare NPs and NP_BSA– exhibited approximately twice the uptake in the GAG-deficient cells compared to the wild-type cells (Figure 7A and 7B). To further validate this finding, we also assessed the uptake of NP_BSA– in enzyme-treated wild-type CHO-K1 cells. Again, increased uptake of NP_BSA– was observed in the glycocalyx-depleted model (Figure 7C). These outcomes imply that the glycocalyx can serve as a barrier to NP internalization in the absence of attractive interactions between NPs and cell-surface GAGs. However, this barrier effect may depend on the specific cell and NP types, as this phenomenon has been observed in some but not all instances.^31,32,35,38,47,71^

Of note, it can be also discerned that NP_BSA– uptake by CHO-K1 cells was higher than that of NP_BSA (compare Figure 6B and Figure 7B). This difference is likely attributed to an efficient scavenger receptor-mediated uptake of NP_BSA–, resulting from the more anionic and partly denatured form of BSA– (see Figure 1D).^23,72^

### Glycocalyx Interactions Enhance the Cellular Uptake of FBS-Coated NPs

As the next step, we prepared FBS-coated NPs (NP_FBS) to investigate how the glycocalyx influences NP uptake in the presence of a more complex protein corona. The results are shown in Suppl. Figures S7 and S6C and discussed in the Supplementary Results. They show that, similar to NP_BSA and NP_BSA+, NPs with an FBS corona exhibit attractive interactions with GAGs, which contribute to enhance NP internalization by cells.

### Cell Uptake of NP_BSA+ Remains Unaffected in Complete Medium

We next examined whether NP uptake would be affected in the presence of serum proteins. This is particularly critical for NP_BSA+, as cationic NPs in serum may be coated by a protein corona and potentially lose their electrostatic interactions with cell-surface GAGs. We conducted NP uptake experiments using CHO-K1 and HeLa cells in culture medium supplemented with 10% FBS. In both cell lines, NP_BSA uptake decreased in the presence of serum, while NP_BSA+ uptake remained unchanged (Suppl. Figure S8). Similarly, a reduction in NP_BSA– uptake was observed in CHO-K1 cells cultured in complete medium. These results suggest that NP_BSA+ could serve as an effective carrier system for intracellular drug delivery applications. This concept is illustrated in the following section.

### Drug-Loaded NP_BSA+ Promotes Increased Cell Death Relative to NP_BSA

**Figure 8:**
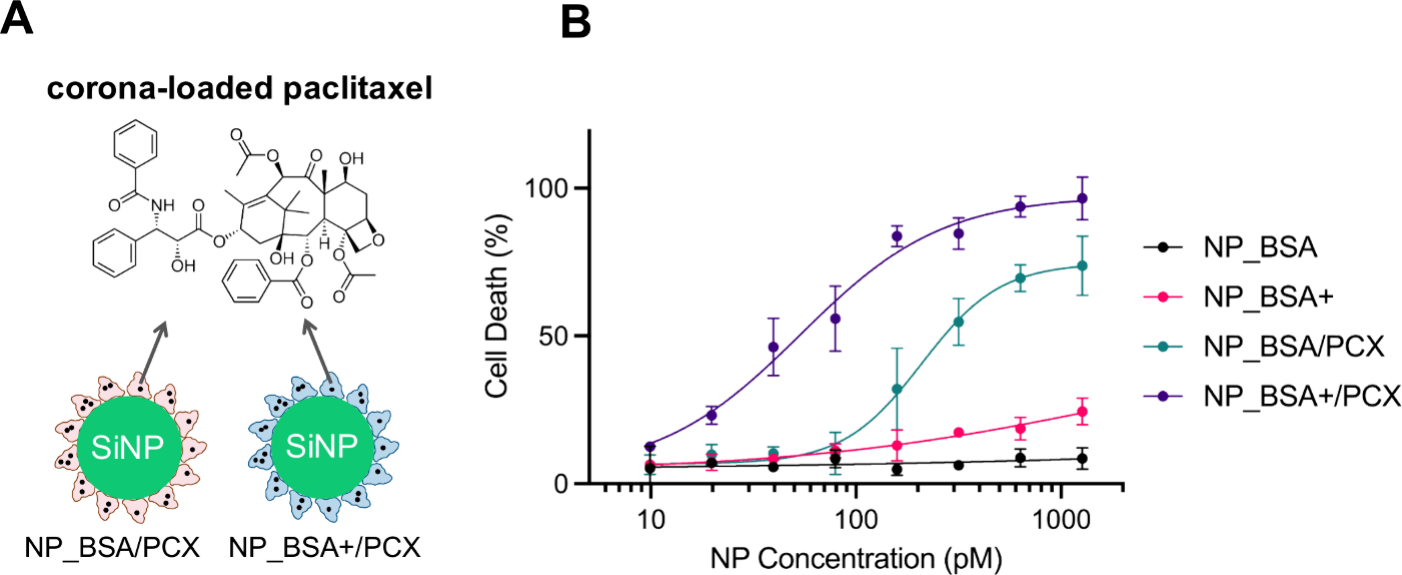
Cytotoxicity in HeLa cells exposed to PCX-loaded NPs. (A) Schematic representations of NP_BSA/PCX and NP_BSA+/PCX. (B) Quantification of cell death in HeLa cells exposed to PCX-loaded NPs (NP_BSA/PCX and NP_BSA+/PCX) and control NPs (NP_BSA and NP_BSA+). Cells were incubated with NPs (0.01–1.3 nM) for 24 h in complete medium, washed to remove excess particles, stained with PI, then analyzed by flow cytometry. Points represent the average of three independent measurements. Curve fitting yielded IC_50_ values of 192 pM for NP_BSA/PCX and 51 pM for NP_BSA+/PCX.

Leveraging ABNP-GAG interactions offers a promising strategy to enhance the cellular uptake of drug-loaded particles and improve therapeutic efficacy. To test this hypothesis, we prepared NP_BSA and NP_BSA+ with the drug paclitaxel (PCX) loaded into the protein coatings, yielding NP_BSA/PCX and NP_BSA+/PCX, respectively (Figure 8A). We then incubated HeLa cells with both control and PCX-loaded NPs in complete medium and quantified the percentage of cell death through the propidium iodide (PI) staining method (Figure 8B). While the control NPs did not cause significant cell death, the PCX-loaded particles induced cell death in a dose-dependent manner. Notably, NP_BSA+/PCX was significantly more potent, with an IC_50_ of 51 pM compared to 192 pM for NP_BSA/PCX. This proof-of-principle demonstration underscores the importance of ABNP-GAG interactions in designing more efficient drug delivery systems.

## Conclusions

ABNPs hold enormous therapeutic potential in the field of cancer drug delivery. In this study, we sought to understand the influence of the glycocalyx on the cellular uptake of albumin-coated silica NPs, including native, cationic, and anionic albumin variants. The results revealed that the glycocalyx enhanced the cell uptake of both NP_BSA and NP_BSA+. The increased uptake was anticipated for NP_BSA+ due to their favorable electrostatic interactions with the glycocalyx. However, the outcome for NP_BSA was surprising, as these particles hold a negative ZP and thereby would be expected to be repelled by the highly anionic glycocalyx. The observed glycocalyx-mediated uptake of NP_BSA could be attributed to attractive BSA-GAG interactions, as demonstrated through experimental and computational analyses using heparin as a model GAG. Presumably, NP_BSA/NP_BSA+ interactions with surface GAGs increase the dwell time and promote the entrapment and retention of the particles near the cell surface, thus improving uptake efficiency. In contrast, NP_BSA– experienced a barrier effect from the glycocalyx, attributable to the absence of attractive BSA-GAG interactions in this case. We also demonstrated how harnessing favorable ABNP-GAG interactions can enhance drug delivery systems. Specifically, we showed that NP_BSA+ loaded with PCX induced significantly more cell death compared to NP_BSA.

Our study has limitations. First, we only investigated a single NP size, while the uptake of larger or smaller particles may be influenced differently by the glycocalyx. Second, the glycocalyx undergoes reorganization in response to shear flow, a condition encountered in vivo.^73−76^ This could result in distinct uptake outcomes compared to those observed here under static conditions. Third, the presence of additional ions, small molecules and proteins in the solution may further modulate NP-GAG interactions and cell uptake.^77^ These aspects warrant further investigation in future studies.

Taken together, our results suggest that strategically exploiting albumin-GAG interactions could offer a novel approach to optimize ABNP systems. For instance, engineering ABNPs with higher affinity for the cancer cell glycocalyx presents an interesting opportunity to enhance endocytosis and therapeutic efficacy in cancer drug delivery. In contrast, ABNPs lacking attractive interactions with surface GAGs may be particularly suited for selective drug delivery to cells with a damaged glycocalyx, which is typical of various pathological conditions, such as atherosclerosis.

## Material and Methods

### Chemicals and Cell Lines

Plain fluorescent silica NPs (Excitation/Emission: 485/510 nm) of 50 nm in diameter were from Kisker Biotech (Germany). BSA, fluorescein isothiocyanate (FITC), FBS, ethylenediamine, citraconic anhydride, MTT reagent, MTX, Ham’s F-12 culture medium, and DMEM culture medium were from Sigma-Aldrich (Brazil). The Micro BCA Protein Assay Kit, PEG-biotin, 1-ethyl-3-(3-(dimethylamino)propyl)carbodiimide hydrochloride (EDC), DMEM/F12 Glutamax, propidium iodide (PI), Alexa Fluor 488-labeled transferrin, FITC-labeled dextran 10 kDa, and FITC-labeled dextran 70 kDa were from Thermo Fisher Scientific (Brazil). Rapigest was from Waters (USA). Unfractionated porcine mucosal heparin (MW ∼ 12 kDa) was from Extrasul (Brazil). FITC-labeled heparin and totally N,O-desulfated heparin were prepared as described in the Supporting Information.^78^ HepIII was from R&D Systems (USA), while Chase was purified from *Flavobacterium heparinum* as described previously.^79^ Anti-HS and anti-CS primary antibodies were from US Biological (USA) and Abcam (USA), respectively, while Alexa Fluor 488 antimouse secondary antibody was from Thermo Fisher Scientific (USA). Wild-type CHO-K1 and the xylosyltransferase-deficient mutant pgsA-745 cell lines were a kind gift from Dr. Jeffrey Esko (University of California at San Diego, USA).^64^

### Preparation and Characterization of Cationic and Anionic Albumin

BSA+ was prepared by reacting native BSA (150 μM) with ethylenediamine (45 mM) in the presence of EDC (5 mM) in MES buffer (0.5 M, pH 4.8). The reaction proceeded with stirring at room temperature for 2 h. BSA– was prepared by mixing native BSA (150 μM) with citraconic anhydride (160 mM) in borate buffer (0.4 M, pH 9) for 2 h. The as-prepared BSA+ and BSA– were purified using a 10 kDa Amicon filter. They were characterized by DLS and ZP measurements in phosphate buffer (10 mM, pH 7.4) employing a Zetasizer Nano ZS instrument (Malvern Instruments, UK). The proteins were also characterized regarding their secondary structure using far-UV CD spectroscopy with a Chirascan Plus instrument (Applied Photophysics, UK). For this purpose, proteins (0.5 μM) dispersed in phosphate buffer were loaded into 1 mm quartz cuvettes and scanned between 200 and 250 nm using a 1 nm bandwidth and 1 nm scan step. Eight repeat scans were accumulated and smoothed using a Savitsky–Golay filter.

### Preparation and Characterization of Albumin-Coated and FBS-Coated NPs

BSA, BSA+ and BSA– (10 mg mL^–1^) were incubated with NPs (3 nM) in PBS buffer (10 mM, pH 7.4, 150 mM NaCl) for 24 h at 4 °C under mild agitation. To ensure complete NP coverage, the molar concentration of protein in solution was approximately 300 times higher than the estimated concentration of adsorbed protein, assuming ideal surface packing. NP coating was then followed by three cycles of centrifugation and washing (16000 g for 30 min at 15 °C in 10% sucrose solution) to remove excess protein. The NPs underwent characterization through DLS, ZP measurements, and CD spectroscopy similarly as described above. FBS-coated NPs were prepared from FBS solutions diluted to 10% by volume in PBS buffer. The remaining procedure was carried out similarly as described for albumin-coated NPs. NP_FBS were characterized by DLS and ZP measurements.

### Determination of the Binding Stoichiometry

The binding stoichiometry, representing the number of adsorbed proteins per NP, was determined using the Micro BSA Protein Assay Kit. Briefly, the various albumin-coated NPs were prepared as described above. Subsequently, the coated NPs were subjected to five cycles of centrifugation and washing to ensure the removal of all excess protein. After the final centrifugation, samples were resuspended in 100 μL of 50 mM NH_4_HCO_3_ buffer containing 25 μL of 0.2% RapiGest, and incubated at 80 °C for 15 min to detach proteins from the NPs. One final centrifugation cycle was performed to remove NPs and collect the supernatant. Protein quantities were then evaluated using the Micro BCA assay. To calculate the binding stoichiometry, we divided the measured protein amounts (in nM) by the corresponding NP concentration (in nM). For the calculation of the theoretical binding stoichiometry, we divided the total NP surface area by the albumin cross-sectional area, yielding ∼200 albumin molecules per NP.

### Mass Spectrometry Analysis of FBS Corona

Liquid chromatography mass spectrometry (LC-MS) experiments were performed in the MSE mode using a Synapt G2 mass spectrometer (Waters) coupled to a nanoAcquity UPLC (Waters).^80,81^ Complete experimental details can be found in the Supporting Information.

### Fluorescence Anisotropy Measurements of Albumin-Heparin Interactions

Albumin-heparin interactions were evaluated by fluorescence anisotropy using a Chirascan Plus instrument equipped with anisotropy accessory. For this purpose, FITC-labeled heparin (250 nM) dispersed in PBS buffer (maintained at pH values of 6.0, 6.4 and 7.4) was titrated with BSA, BSA+, and BSA–. Anisotropy measurements were recorded using an excitation wavelength of 495 nm and a 515 nm long-pass filter to collect the emission signal. The following equation was used to fit the data, from which an estimate of the apparent *K*_D_ was obtained:^82^*r* = *r*_0_ + (*r*_b_ – *r*_0_) × *L*/(*L* + *K*_D_), where *r* is the measured anisotropy, *r*_0_ is the initial anisotropy, *r*_b_ is the anisotropy at saturation, and *L* is the concentration of ligand.

### Computational Modeling and Dynamics Simulations

Details are available in the Supporting Information.

### Characterization of NP-Heparin Interactions

NP-heparin interactions were initially assessed using DLS. NP_BSA, NP_BSA+, NP_BSA–, and NP_FBS (0.63 nM) were incubated with increasing concentrations of heparin in PBS buffer. The resulting NP aggregation induced by heparin was monitored by DLS. Additionally, the interactions were characterized using MST with a Monolith NT.115 instrument (Nanotemper Technologies, Germany). NP_BSA and NP_FBS (1 nM) were titrated with heparin in PBS buffer. Binding curves were generated by monitoring changes in thermophoresis as a function of heparin concentration. Data was converted to “Fraction bound” in the MST software and used as such for analysis and presentation. SPR measurements of NP-heparin interactions were performed using a Biacore T-200 instrument (Cytiva, Sweden). For this purpose, biotinylated heparin was immobilized onto streptavidin-coated sensor surfaces at a density of 150 RU. In addition, PEG-biotin was immobilized onto the remaining streptavidin binding sites on the reference surface to minimize nonspecific interactions with NPs. NP_BSA+ dispersed in PBS buffer was injected into the flow at concentrations ranging from 0.00625 to 0.2 nM, and the flow rate was 60 μL min^–1^. The association and dissociation phases were recorded for 200 and 900 s, respectively. Regeneration of the sensor surface between injections was achieved with sodium dodecyl sulfate in water (0.01%), followed by a solution of NaCl in water (2 M) (injection time = 60 s and flow rate = 30 μL min^–1^). Bulk refractive index changes were corrected for by subtracting the signal response of the reference surface from the raw SPR traces. Data analysis was carried out with the software EVILFIT, assuming a continuous distribution of equilibrium constants and kinetic rate constants.^83^

### Cytotoxicity tests

Details are available in the Supporting Information.

### Cellular Uptake of NPs

CHO-K1 and pgsA-745 cells were cultured in Ham’s F-12 culture medium containing 10% FBS and 1% penicillin/streptomycin at 37 °C in a humidified 5% CO_2_ atmosphere. Cell culture was restricted to passages 1–7 after defrosting. For NP uptake experiments, cells were seeded in 24-well plates (∼7.5 × 10^4^ cells per well) and grown to approximately 70% confluence. Cells were incubated with NPs (3 nM) for 4 h at 37 °C in serum-free F-12 culture medium. The various NP types employed in these experiments included NP_BSA, NP_BSA+, NP_BSA– and NP_FBS, alongside uncoated NPs (at 0.16 nM to prevent cytotoxicity). Following the incubations, cells were washed three times with ice-cold PBS, detached with PBS-EDTA 0.05%, and loaded in the flow cytometer. Flow cytometry analysis was performed using a BD Accuri C6 instrument (BD Technologies, USA) equipped with a 488 nm laser. Approximately 10,000 cell events were collected following the application of appropriate gating strategies to remove cell debris. Results were expressed as median fluorescence intensities of fluorescence-intensity distributions.

NP uptake by CHO-K1 cells was further evaluated under specific conditions, as follows: (i) Cells were treated with HepIII (120 mU mL^–1^), Chase (1.0 μL mL^–1^), or a combination of both enzymes for 2h at 37 °C. Next, NPs were added to the culture medium, and uptake experiments were conducted for 2h; (ii) Cells were incubated with heparin or totally N,O-desulfated heparin (100 μg mL^–1^) for 2 h, followed by treatment with NPs for an additional 2h. (iii) Cells were treated with MTX (50 μM) in culture medium for 30 min and then incubated with NPs for an additional 2 h. The remaining procedure was performed as described above.

The uptake of NP_BSA, NP_BSA+, and NP_FBS was also investigated in HeLa cells. Cells were cultured in DMEM culture medium supplemented with 10% FBS and 1% penicillin/streptomycin. Uptake was assessed in pristine cells and cells treated with HepIII and Chase, as well as in the presence of excess heparin. The cells were incubated with NPs (0.63 nM) for 2h at 37 °C in serum-free culture medium, and then processed as described above.

Finally, the uptake of NP_BSA, NP_BSA+, and NP_BSA– by CHO-K1 and HeLa cells was evaluated in complete medium containing 10% FBS. For this purpose, NPs were first dispersed in both serum-free and complete medium for 30 min. The NPs in their respective media were then incubated with the cells for 4 h at 37 °C. The remaining procedure was implemented as previously described.

### Preparation of PCX-Loaded NPs and Cell Death Assessment in HeLa Cells

BSA and BSA+ dispersed in 1.8 mL of phosphate buffer solution ([BSA] = 150 μM) were mixed with 0.2 mL of PCX in ethanol ([PCX] = 11 mM). The resulting solution was rotated overnight, after which it was centrifuged (16,000 g for 20 min) to precipitate and remove unbound PCX present as large aggregates in solution. Subsequently, NPs coated with both PCX-loaded BSA and BSA+ were prepared and purified similarly as described above. It was previously reported that a single albumin molecule can bind up to six PCX molecules under conditions of large PCX excess.^84^ Therefore, given that each NP is coated with approximately 200 albumin molecules, we can estimate a PCX concentration of around 1 μM for a 1 nM NP solution. HeLa cells in complete medium were then incubated with the drug-loaded NPs at varying concentrations (0.01–1.3 nM) for 24 h. Following this, the cells were washed, stained with PI, and analyzed by flow cytometry as outlined earlier. The cell death results were plotted as a function of log[NP] and analyzed using GraphPad Prism to determine the IC_50_ values through curve fitting.

### Statistical Analysis

To assess statistical significance in the cell uptake experiments, unpaired *t* tests were performed for comparisons between two groups, and ANOVA was performed for comparisons between three or more groups, accompanied by Bennet posthoc tests for pairwise comparisons. All data was tested for homogeneity of variances (Levenne’s test) and normality (Shapiro-Wilk test and Q-Q plot evaluation) prior to statistical tests. Results were considered statistically significant if *p* < 0.05, with *p < 0.05, **p < 0.01, ***p < 0.001, and **** *p* < 0.0001. Each cell uptake condition was evaluated in at least three independent experiments, each performed in technical triplicate.

## Supporting Information Available

Supporting Information and Methods; Supplementary Results on the cell uptake of FBS-coated NPs; Cytotoxicity tests; Comparison of NP uptake at 4 and 37 °C; GAG labeling in pristine and enzyme-treated cells; Influence of mitoxantrone on the cell uptake of cationic NPs and endocytic markers; NP uptake in control and enzyme-treated HeLa cells; Characterization of FBS-coated NPs, interactions with heparin, and NP uptake; Comparison of NP uptake in serum-free and complete media (PDF)
