# Supplementary material for: Glycocalyx Interactions Modulate the Cellular Uptake of Albumin-Coated Nanoparticles
Source: ACS Appl Bio Mater. 2024 Oct 29;7(11):7365–77. doi: 10.1021/acsabm.4c01012 (PMC11577421; doi:10.1021/acsabm.4c01012)
Supplement: Supplementary file 1 — mt4c01012_si_001.pdf [file mt4c01012_si_001.pdf]

Supporting Information

Glycocalyx Interactions Modulate the Cellular Uptake of Albumin-Coated Nanoparticles

Paulo H. Olivieri Jr,[1,#] Isabela F. Assis,[1,#] Andre F. Lima,[1] Sergio A. Hassan,[2] Ricardo J.S. Torquato,[1] Jackelinne Y. Hayashi,[1] Alexandre K. Tashima,[1] Helena B. Nader,[1] Anna Salvati,[3] Giselle Z. Justo,[1,*] Alioscka A. Sousa[1,*]

1. Department of Biochemistry, Federal University of São Paulo, São Paulo, SP 04044-020, Brazil

2. Bioinformatics and Computational Biosciences Branch, National Institute of Allergy and Infectious Diseases, National Institutes of Health, Bethesda, MD 20892, USA

3. Department of Nanomedicine & Drug Targeting, Groningen Research Institute of Pharmacy (GRIP), University of Groningen, Groningen, The Netherlands

[#] These authors contributed equally to this work

* Corresponding authors: giselle.zenker@unifesp.br; alioscka.sousa@unifesp.br

SUPPLEMENTARY MATERIALS AND METHODS

Computational modeling and dynamics simulations

Initial coordinates of the heparin disaccharide, Hd (2-O-sulfo-α-L-idopyranuronic acid-(1-4)-2-deoxy-6-O-sulfo-2-(sulfoamino)-α-D-glucopyranose), were extracted from the NMR structure PDB ID 1HPN; coordinates for heparan monosulfated disaccharide (HS1d) and heparan diphosphate disaccharide (HS2d) were based on Hd with the appropriate modifications (cf. inset of Figure 3). Molecular dynamics (MD) simulations were carried out with NAMD using the CHARMM param36 force field; parameters for Hd and HDs were based on existing topology and parameter files in the CHARMM distribution package (c46b1). Initial coordinates of human serum albumin were obtained from the crystal (PDB: 1AO6). For comparative analysis, all simulations were performed under identical conditions of temperature (25 °C) and pressure (1 atm), salt concentration (120 mM of NaCl), and standard protonation states at neutral pH (calculated with the PropKa software; Arg^+ and Lys^+, Glu^- and Asp^-, and neutral His, yielding an overall charge of -15 e). A single albumin and twenty disaccharides were introduced in a cubic box with a side length of 94 Å filled with pre-equilibrated TIP3P water molecules. Periodic boundary conditions were applied to the three dimensions, and particle mesh Ewald summations were used to calculate long-range forces. Ten independent simulations of 0.5 μs each (counted after thermal equilibration was attained) were performed, with each simulation starting with random distributions of the solutes. Statistics were collected during the last 0.4 μs and aggregated in a single data file for analysis. Albumin-disaccharide interactions were calculated based on a distance (δ) criterion (soft definitions) from the trajectory: polar and nonpolar interactions are based on the distance between sidechain donor and acceptor atoms ($\delta_{AD} < 3$ Å) and sidechain carbon atoms ($\delta_{CC} < 4.8$), respectively. The frequency of interactions of residue X with the disaccharide molecules throughout the simulation was considered a proxy for the strength of interactions of X with the disaccharide subsystem. The corresponding normalized statistics were mapped as heatmaps on each interacting residue and listed in the bfactor column of the PDB coordinates files.

Mass spectrometry analysis

NP_FBS were prepared as described for the albumin-coated NPs, with two additional centrifugation cycles to ensure the removal of excess protein. After the final centrifugation, samples were resuspended in 100 μL of 50 mM NH_4HCO_3 buffer containing 25 μL of 0.2% RapiGest (Waters, United States), and incubated at 80 °C for 15 min to detach proteins from the NPs. One final centrifugation cycle was performed to remove NPs and collect the supernatant. Protein quantities were then evaluated by Micro BCA assay. For protein digestion, samples were treated with 5 mM DTT at 60°C for 30 min, followed by 10 mM IAA at 25 °C for 30 min in the dark. Trypsin was then added to the samples (1:100 enzyme/protein) and incubated overnight. Digestion was interrupted by adding 5% TFA, and the samples were dried. Peptides were fractioned using StageTip C18 columns to remove excess salts and quantified via the Pierce Quantitative Colorimetric Peptide Assay (Thermo Fisher Scientific, Brazil), following the manufacturer's protocol. Liquid chromatography mass spectrometry (LC-MS) experiments were performed in the MSE mode using a Synapt G2 mass spectrometer (Waters) coupled to a nanoAcquity UPLC (Waters). Aliquots of 2 μg were injected into a trap column (Acquity UPLC M-Class Symmetry C18 Trap Column, 100 Å, 5 μm, 300 μm x 25 mm, Waters), loaded with phase A (0.1% formic acid) at a flow rate of 8 μL/min for 3 min, and then eluted to an analytical column (Acquity UPLC M-Class HSS T3 Column, 1.8 μm, 300 μm ×150 mm,Waters) using an elution gradient of 7–35% B (0.1% formic acid in ACN) in 60 min at 3 μL/min. Peptide ions were fragmented by collision-induced dissociation (CID), and energies were alternated between 4 eV and a ramp of 23−55 eV for precursor and fragment ions, respectively, using scan times of 0.75 s. Runs were performed in triplicate. LC-MS data were processed in Progenesis QI for proteomics (PQIP, Nonlinear Dynamics). Database search was performed using the Bos taurus database (57,591 sequences, downloaded from UniProt on June 1st, 2024), utilizing de novo sequenced peptides and a maximum false discovery rate of 1%. Relative quantification was performed using the average MS signal response of the three most intense tryptic peptides of each protein

Preparation of totally N,O-desulfated heparin

Totally N,O-desulfated heparin was prepared from parental unfractionated porcine mucosal heparin as previously described.[1] Briefly, 200 mg of the pyridinium salt of heparin underwent exhaustive desulfation in a solution of DMSO with 10% methanol at 105 °C overnight. Afterwards, the pH was adjusted to 9.0 with NaOH, the solution was cooled, and the product was precipitated with ethanol saturated with sodium acetate. The precipitates were collected by filtration, washed with cold ethanol, and dissolved in a small volume of deionized water. The modified heparin was purified by gel filtration chromatography using the ÄKTA Avant system (GE Healthcare, USA) equipped with a HiPrep 26/10 desalting column (Sephadex G-25; Cytiva, Sweden) and eluted with deionized water. Chemical modifications were confirmed using 1H and heteronuclear single quantum coherence NMR spectra at 500 MHz with a Bruker Ascend spectrometer (Karlsruhe, Germany) in D_2O as previously described.[1,2]

Preparation of FITC-labeled heparin

For the functionalization of heparin with FITC, 100 µL of a FITC solution (1 mg/mL) in dimethyl sulfoxide (DMSO) was slowly added to 1 mL of a heparin solution (2 mg/mL) in sodium carbonate buffer (0.1 M, pH 9.0). The mixture was incubated overnight at room temperature in the dark. The resulting FITC-heparin conjugate was purified by gel filtration chromatography using a PD-10 desalting column (Sephadex G-25, Cytiva, Sweden). The final product was visualized by agarose gel electrophoresis in propylene-diamine-acetate buffer (0.05 M, pH 9.0) using a Typhoon FLA 9500 (GE Healthcare, Sweden) as previously described.[3]

Cytotoxicity tests

For the MTT cytotoxicity assay, cells were seeded in 96-well plates (~10^4 cells per well) the day before the experiments and then incubated with various concentrations of NPs for 4 h. Following incubation, the media was removed and replaced with MTT reagent (100 µL, 0.5 mg mL^{-1}) in F-12 medium without phenol red, in accordance with the manufacturer's protocol. After 3 hours, cells were solubilized with 10 µL of DMSO, and absorbance was measured at 590 nm using a Spectramax 684 plate reader (Molecular Devices, United States). Unseeded wells served as the blank, and untreated cells were used as a viability control. A similar procedure was

used to evaluate the cytotoxicity of $NaClO_3$ and MTX following their incubation with cells for 24 and 2 h, respectively. For the PI assay, cells were seeded in 24-well plates similarly as described above and then treated for 4 h with the same NP concentrations as used in the uptake studies. Cells were detached and analyzed using a flow cytometer. After fluorescence compensation, PI-positive cells were gated, and the percentage of cell death was determined.

SUPPLEMENTARY RESULTS

Glycocalyx interactions enhance the cellular uptake of FBS-coated NPs

We prepared FBS-coated NPs (NP_FBS; **Suppl. Figure S7A**) to investigate how the glycocalyx influences NP uptake in the presence of a more complex protein corona. These experiments aimed to gather further evidence that favorable interactions between protein-coated NPs and GAGs can enhance NP uptake. The size and ZP of NP_FBS are included in **Table 1**, while the identity of the major adsorbed FBS proteins, determined using mass spectrometry, is depicted in **Suppl. Figure S7B**. The top 15 adsorbed proteins, which accounted for 90% of the total adsorbed protein mass, included albumin alongside well-known heparin-binding proteins such as apolipoprotein A-1, apolipoprotein E, complement factor H, and factor XII.[4-6] Therefore, it is conceivable that NP_FBS may interact with surface GAGs through more specific and high-affinity interactions compared to NP_BSA, although a detailed examination of these interactions was beyond our scope.

To verify the occurrence of general GAG-corona interactions, NP_FBS were titrated with heparin and analyzed via DLS (**Suppl. Figure S7C**). The results indicated the formation of large aggregates induced by heparin, suggesting binding to the FBS corona. MST analysis further confirmed this interaction, yielding an apparent K_D of about 165 µM (**Suppl. Figure S7D**).

The cell uptake of NP_FBS was evaluated in a manner similar to the albumin-coated NPs. Uptake experiments were conducted with CHO-K1 and pgsA-745 cells (**Suppl. Figure S7E**) as well as CHO-K1 cells treated with GAG-cleaving enzymes (**Suppl. Figure S7F**), heparin/desulfated heparin (**Suppl. Figure S7G**), and MTX (**Suppl. Figure S7H**). The results indicated that cell uptake was greater when GAG-

corona interactions were left intact, with the exception of CHO-K1 cells treated with MTX, which showed no difference in uptake relative to control cells. Additionally, we evaluated NP uptake in pristine and enzyme-treated HeLa cells, along with HeLa cells treated with excess heparin (**Suppl. Figure S6C**). It can be seen that NP uptake decreased following cell treatment with a mixture of HepIII and Chase. In the presence of excess heparin, a trend of reduced NP uptake was observed, albeit without statistical significance.

Collectively, these findings suggest that NPs covered with an FBS corona exhibit attractive interactions with GAGs, which contribute to enhance NP internalization by cells.

SUPPLEMENTARY TABLE AND FIGURES

Table S1. Effect of NP exposure on cell viability measured by propidium iodide (PI) staining and flow cytometry. NP concentrations were fixed at the same levels used in uptake studies.

PI positive cells (%)		
SiNP	CHO-K1	pgsA-745
Control	0.9 ± 0.02	1.34 ± 0.1
Bare	7.34 ± 3.51	9.45 ± 0.75
NP_BSA	1.97 ± 0.27	1.31 ± 0.41
NP_BSA+	2.5 ± 0.92	1.87 ± 0.56
NP_BSA−	1.49 ± 0.56	1.98 ± 0.81
NP_FBS	2.58 ± 0.68	2 ± 0.55

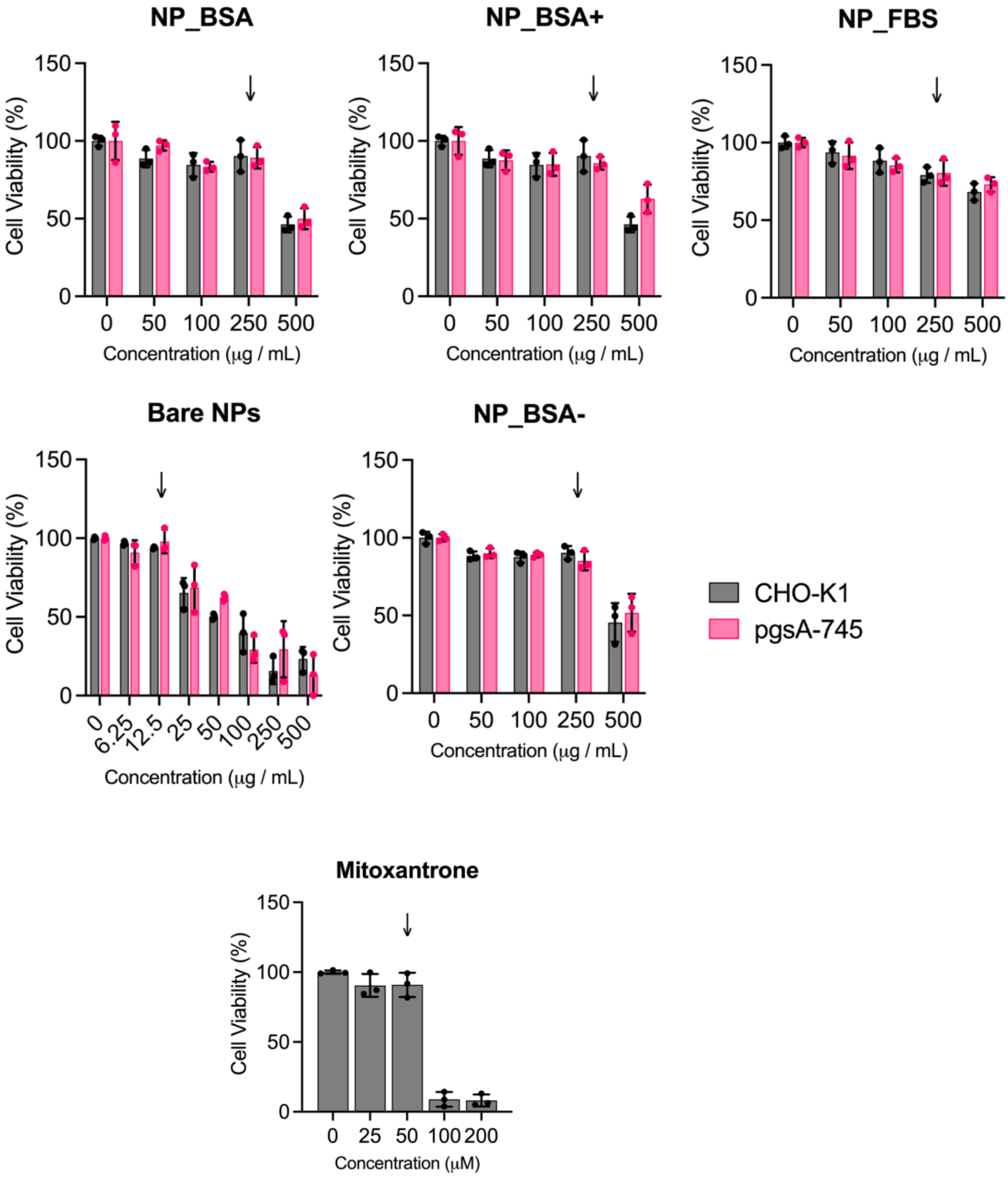

Figure S1. Cytotoxicity tests. Effect of exposure to NPs and treatment with mitoxantrone on cell viability measured by MTT assay. Arrows indicate the actual concentrations used in uptake studies.

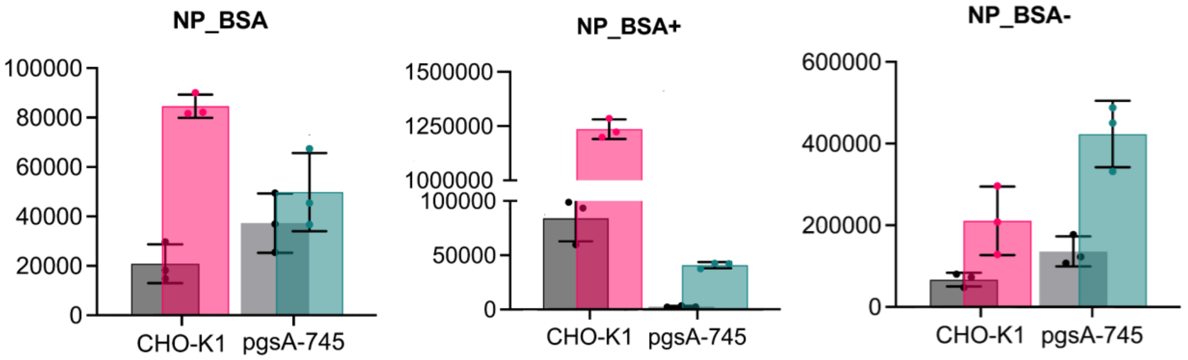

Figure S2. Comparison of albumin-coated NP uptake by CHO-K1 and pgsA-745 cells at 4 °C (gray bars) versus 37 °C (pink and green bars). Cells were incubated with NPs (3 nM) for 4 h in culture medium at either 4 or 37 °C, washed to remove excess particles, and then analyzed by flow cytometry. Except for NP_BSA uptake by pgsA-745 cells, the significantly higher uptake levels at 37 °C compared to 4 °C are indicative of active NP endocytosis.

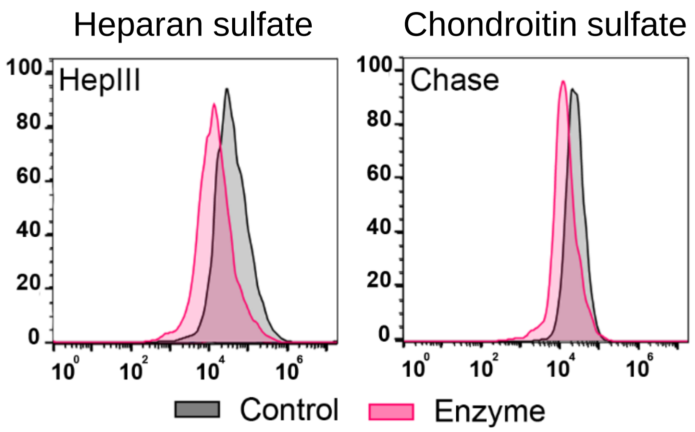

Figure S3. Characterization of HS and CS content in pristine and enzyme-treated CHO-K1 cells. Cells were labeled with either anti-HS or anti-CS primary antibodies followed by a fluorescent secondary antibody. Subsequently, cells were analyzed by flow cytometry. Please refer to ref. [7] for additional details.

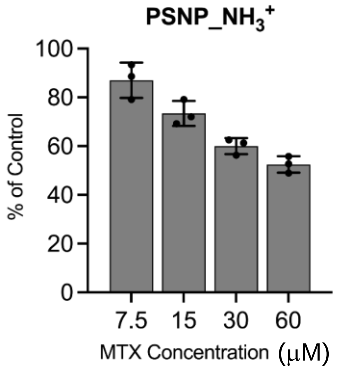

Figure S4. Effect of mitoxantrone (MTX) on the uptake of cationic polystyrene NPs (PSNP_NH_3^+) by CHO-K1 cells. Cells were pretreated with MTX for 30 min in culture medium. Subsequently, cells were incubated with PSNP_NH_3^+ (200 μg/mL) for an additional 1 h. Cells were then washed with ice-cold PBS and analyzed by flow cytometry.

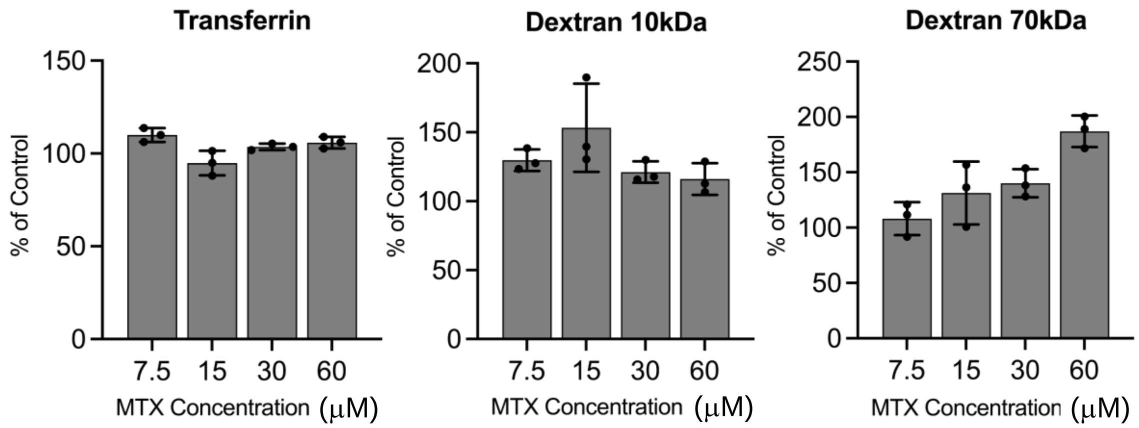

Figure S5. Effect of mitoxantrone (MTX) on distinct endocytic pathways in CHO-K1 cells. Endocytic markers included: FITC-labeled dextran 10 kDa, a general fluid-phase marker incorporated into all forming vesicles; FITC-labeled dextran 70 kDa, a selective marker for macropinocytosis; Alexa Fluor 488-labeled transferrin, a classical cargo for clathrin-mediated endocytosis.[8] Cells were pretreated with MTX for 30 min in culture medium. Subsequently, cells were washed and then incubated with transferrin (50 μg/mL), dextran 10 kDa (200 μg/mL), or dextran 70 kDa (200 μg/mL) for 5 min (transferrin) or 30 min (dextran) in culture medium. Cells were then washed with ice-cold PBS and analyzed by flow cytometry.

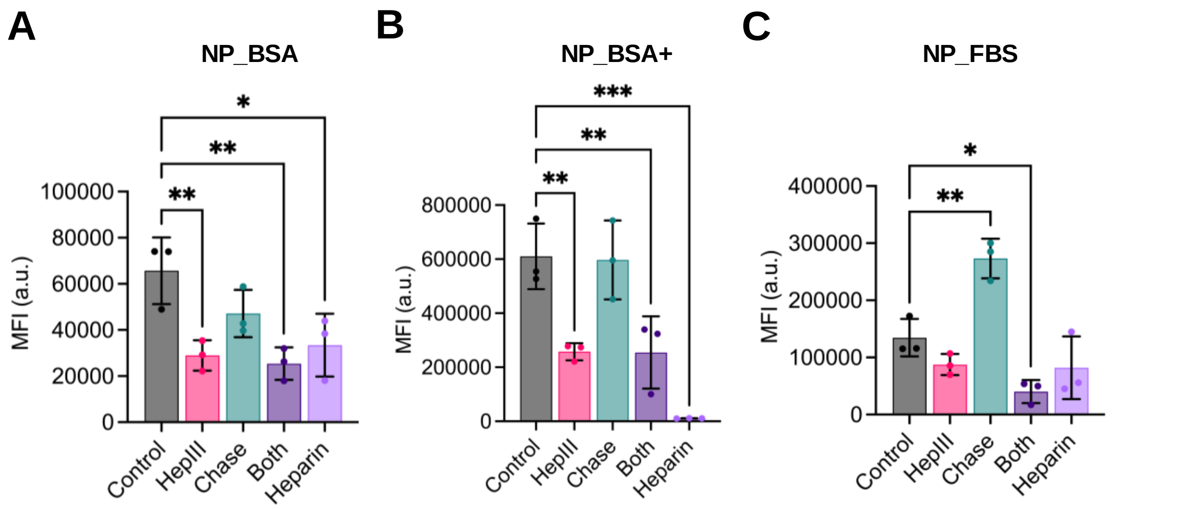

Figure S6. Uptake of (A) NP_BSA, (B) NP_BSA+, and (C) NP_FBS by HeLa cells. Cells were pretreated with buffer (Control), glycosidic enzymes, or excess heparin before NP administration for 2 h in culture medium. Next, cells were washed to remove excess particles and analyzed by flow cytometry.

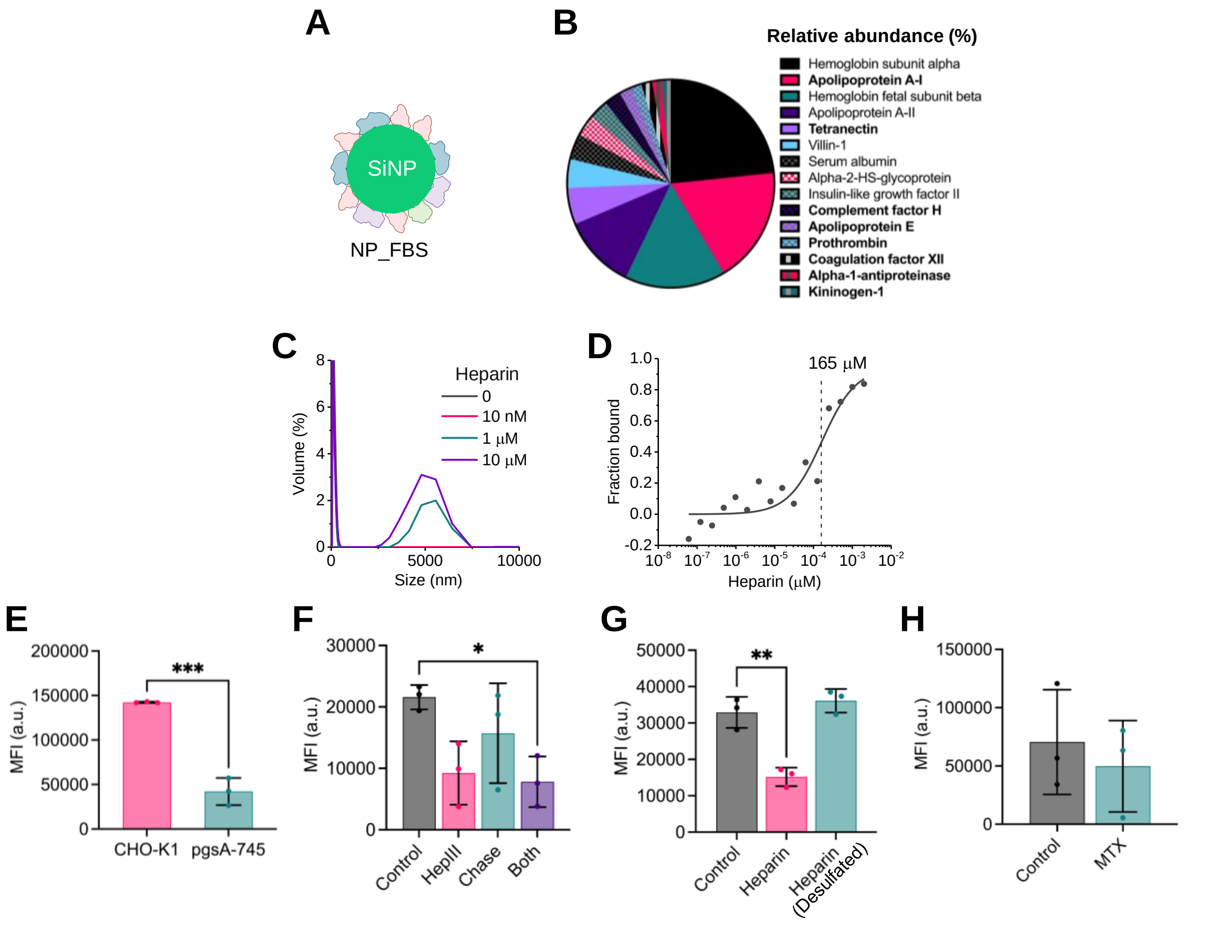

Suppl. Figure S7. Characterization of NP_FBS, interactions with heparin, and uptake by CHO cells. (A) Schematic illustration of NP_FBS. (B) Adsorbed FBS proteins identified by mass spectrometry. Heparin-binding proteins are highlighted in bold. (C) Dynamic light scattering measurements of NP_FBS titrated with heparin. Heparin interactions bridged NPs, leading to their aggregation. (D) Microscale thermophoresis measurements of NP_FBS titrated with heparin. Points represent the average of three measurements. The apparent binding affinity (K_D) is indicated in the plot. (E) NP uptake by CHO-K1 and pgsA-745 cells. Cells were incubated with NP_FBS for 4 h in culture medium, washed to remove excess particles, then analyzed by flow cytometry. (F) NP uptake by CHO-K1 cells treated with HepIII, Chase, and both. (G) NP uptake by CHO-K1 cells treated with excess heparin and desulfated heparin. (H) NP uptake by CHO-K1 cells treated with MTX. In (F-H), CHO-K1 cells were pretreated with glycosidic enzymes, excess heparin, or chemical inhibitors before NP administration for 2 h in culture medium.

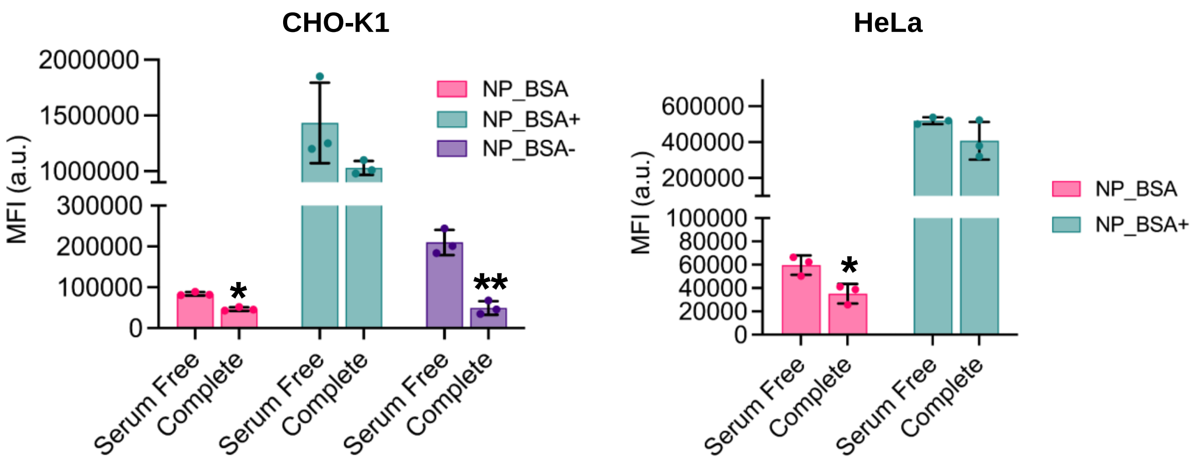

Figure S8. Uptake of albumin-coated NPs by CHO-K1 and HeLa cells in both serum-free culture medium and serum-containing medium (Complete). Cells were incubated with NPs for 4 h in the corresponding media, washed to remove excess particles, then analyzed by flow cytometry.

1. Yates, E. A.; Santini, F.; Guerrini, M.; Naggi, A.; Torri, G.; Casu, B. 1H and ^{13}C NMR spectral assignments of the major sequences of twelve systematically modified heparin derivatives. *Carbohyd. Res*. 1996, 294, 15-27.

2. Meneghetti, M. C.; Naughton, L.; O'Shea, C.; Koffi Teki, D. S.-E.; Chagnault, V.; Nader, H. B.; Rudd, T. R.; Yates, E. A.; Kovensky, J.; Miller, G. J.; Lima, M. A. Using NMR to Dissect the Chemical Space and O-Sulfation Effects within the O-and S-Glycoside Analogues of Heparan Sulfate. *ACS Omega* 2022, 7 (28), 24461-24467.

3. Nader, H. B.; Buonassisi, V.; Colburn, P.; Dietrich, C. P. Heparin stimulates the synthesis and modifies the sulfation pattern of heparan sulfate proteoglycan from endothelial cells. *J. Cell. Physiol*. 1989, 140 (2), 305-310.

4. Gómez Toledo, A.; Sorrentino, J. T.; Sandoval, D. R.; Malmström, J.; Lewis, N. E.; Esko, J.D. A Systems View of the Heparan Sulfate Interactome. *J. Histochem. Cytochem.* 2021, 69 (2), 105-119.

5. Gesslbauer, B.; Derler, R.; Handwerker, C.; Seles, E.; Kungl, A. J. Exploring the glycosaminoglycan–protein interaction network by glycan-mediated pull-down proteomics. *Electrophoresis* 2016, 37 (11), 1437-1447.

6. Peysselon, F.; Ricard-Blum, S. Heparin–protein interactions: from affinity and kinetics to biological roles. Application to an interaction network regulating angiogenesis. *Matrix Biol.* 2014, 35, 73-81.

7. Olivieri, P. H.; Jesus, M. B.; Nader, H. B.; Justo, G. Z.; Sousa, A. A. Cell-surface glycosaminoglycans regulate the cellular uptake of charged polystyrene nanoparticles. *Nanoscale* 2022, 14 (19), 7350-7363.

8. Rennick, J. J.; Johnston, A. P.; Parton, R. G. Key principles and methods for studying the endocytosis of biological and nanoparticle therapeutics. *Nat. Nanotech.* 2021, 1-11.
